# Outcomes of myocardial revascularization in patients with obesity and multivessel coronary artery disease

**DOI:** 10.1186/s43044-024-00548-5

**Published:** 2024-08-28

**Authors:** Maira I. Madiyeva, Marat A. Aripov, Alexey Y. Goncharov, Rakhima Y. Zholdasbekova

**Affiliations:** 1 Department of Emergency Cardiology, Pavlodar Regional Cardiology Center, 10/3 Tkacheva St, Pavlodar, 140000 Kazakhstan; 2 Department of Therapy, Semey Medical University, Semey, Kazakhstan; 3 Department of Interventional Cardiology, National Research Cardiac Surgery Center, Astana, Kazakhstan

**Keywords:** Coronary artery bypass grafting, Multivessel coronary artery disease, Obesity, Percutaneous coronary intervention, SYNTAX score

## Abstract

**Background:**

There is uncertainty regarding the best revascularization approach—whether coronary artery bypass grafting (CABG) or percutaneous coronary intervention (PCI)—for obese patients suffering from multi-vessel coronary artery disease.

**Results:**

406 patients with low and intermediate SYNTAX scores (SS) underwent PCI with drug-eluting stents (n = 200, 100 with SS ≤ 22, and 100 with SS 23–32) and CABG (n = 206, 100 with SS ≤ 22, and 106 with SS 23–32). Patients were also categorized by body mass index (BMI): normal weight (12%, 48 patients), overweight (41.6%, 169 patients), and obese (46.6%, 189 patients). The follow-up period averaged 9 ± 1.9 years. The endpoints of the study were as follows: major adverse cardiac and cerebrovascular events, a repeat revascularization, diminished left ventricular ejection fraction (LVEF), and high SS (≥ 33) observed over time. When comparing PCI and CABG in overweight individuals, the risk of myocardial infarction (MI) following PCI was greater than after CABG (Hazard Ratio [HR] 2.7, 95% Confidence Interval [CI] 1.1–6.7, *p* = 0.03). In patients with overweight and Class I obesity, CABG was associated with the risk of coronary atherosclerosis progression (SS ≥ 33) (HR 4.4, 95% CI 1.5–13, *p* = 0.009 and HR 4.9, 95% CI 1.9–12, *p* = 0.001, respectively); whereas PCI was connected with the likelihood of repeat revascularization (HR 2.7, 95% CI 1.6–4.55, p < 0.0001 and HR 2, 95% CI 1.3–3.1, *p* = 0.002, respectively). At the same time, for stented patients, Class III obesity was associated with the risk of repeat revascularization (HR 2.5, 95% CI 1.02–6, *p* = 0.044).

**Conclusion:**

There were no significant weight-related impacts on long-term outcomes among patients who underwent surgery. Whereas in stented patients, Class III obesity was associated with the risk of repeat revascularization. When comparing PCI and CABG, for overweight and Class I obesity patients, CABG was associated with a likelihood of coronary atherosclerosis progression (SS ≥ 33), while PCI was linked to the risk of repeat revascularization. For overweight patients, CABG outperformed PCI in terms of the risk of MI. For other adverse events in patients of different weight categories, PCI and CABG did not reveal any significant benefits.

## Background

Despite the decline in mortality from cardiovascular diseases (CVD) over the past decades, coronary artery disease (CAD) continues to be the primary cause of death globally [1, 2]. Obesity is a strong independent and mediated risk factor for CVD [3, 4] and is also closely associated with coronary atherosclerosis [5, 6]. At the same time, the incidence of obesity has surged to epidemic levels [7]. In this setting, healthcare providers are encountering an increasing number of individuals receiving treatment with distinct clinical characteristics and facing challenges in medical, interventional, and surgical treatments. Historically, studies have shown that coronary artery bypass grafting (CABG) generally surpasses percutaneous coronary intervention (PCI) in various metrics, including survival rates, for patients with multivessel CAD [8–10]. It would be logical to assume that surgical treatment of CAD is beneficial for obese patients. Nonetheless, the advent of advanced drug-eluting stents (DES) has brought into question the applicability of earlier findings to present-day practices. Recent large-scale studies have shown minimal differences in outcomes between PCI and CABG [11–13]. Also, when choosing a strategy, it is necessary to take into account the anthropometric characteristics of this cohort of patients, the high invasiveness of CABG, in comparison with PCI. At the same time, data on the impact of obesity on the success of PCI and CABG are becoming obsolete [14–16], current studies are presented to a greater extent for interventional revascularization than for surgery [17–21]. Therefore, this study's examination of the extended results of PCI using DES compared to CABG in obese patients with multivessel CAD is both pertinent and necessary.

## Methods

### Study design and patients

This investigation was designed as a longitudinal, retrospective clinical cohort two-central study and was described earlier [22]. Briefly, according to archival data from two hospitals, we identified 406 patients who had stable multivessel CAD exhibiting low to intermediate coronary atherosclerotic damage as per the SYNTAX score (SS) (< 33 points) (https://syntaxscore2020.com) [23, 24]. The selected patients underwent initial PCI with DES (200 patients, 100 with SS ≤ 22, and 100 with SS 23–32) and initial CABG (206 patients, 100 with SS ≤ 22, and 106 with SS 23–32) between 2010 and 2013. SS assessments were not primarily utilized but were subsequently applied to archival angiograms in a retrospective manner. Exclusion criteria included previous stenting or cardiac surgery, single-vessel coronary disease, left main disease, an SS ≥ 33, an acute coronary syndrome with an ST elevation, age over 65, left ventricular aneurysm, severe valvular dysfunction combined with CAD, a left ventricular ejection fraction (LVEF) below 40%, rheumatic or congenital heart defects, and severe chronic renal failure (i.e., a glomerular filtration rate [GFR] < 30 ml/min/1.73 m2 using the Cockcroft-Gault equation). Patients were tracked using the data of clinical electronic databases of centers, data from national electronic polyclinic and inpatient registers (https://pvd.dmed.kz, www.eisz.kz), and up-to-date contact information. The average follow-up spanned 9 ± 1.9 years; with the longest follow-up being 12 years.

Moreover, participants in the study were stratified by weight gradations according to their body mass index (BMI), determined by dividing weight in kilograms by height in meters squared (kg/m^2^). These categories were classified into normal (BMI = 18.5–24.9), overweight (BMI = 25–29.9), and obese (BMI ≥ 30) as per World Health Organization definitions. Further, obesity was subclassified into Class I (BMI = 30–34.9), Class II (BMI = 35–39.9), and Class III (BMI ≥ 40) [25, 26].

Following the Helsinki Declaration's principles, the study was approved by the ethics committees of the participating centers.

### Study endpoints

The clinical outcomes targeted in this study included a combined measure of major adverse cardiac and cerebrovascular events (MACCE) and their individual elements: all-cause mortality, cardiac death, cerebrovascular accidents (CVA), which encompass transient ischemic attacks (TIA) and strokes, myocardial infarction (MI); repeated revascularization, and the development of chronic heart failure (CHF). CHF was assessed in accordance with clinical evaluations, measurement of LVEF; and examination for dilatation of the heart chambers with valvular dysfunction. Additionally, high-grade coronary artery lesions, characterized by a SYNTAX score of ≥ 33 observed over time, were also monitored. Deaths were classified as cardiovascular unless a definitive non-cardiovascular cause could be confirmed.

### Statistical analysis

Groups were stratified and evaluated by weight category. The analysis of continuous variables was performed using either univariate analysis of variance (ANOVA) or the Kruskal–Wallis test, contingent upon how the data was distributed. The Chi-square test or the Kendall-Stewart test was used to compare categorical variables, which were expressed as proportions and figures. Evaluation of distant events during the observation period was executed via the Kaplan–Meier method with the log-rank test. The hazard ratio (HR) with a 95% confidence interval (CI) was estimated based on Cox proportional regression. This multivariate analysis aimed to determine whether BMI serves as an independent predictor of adverse outcomes. Relevant covariates included in the Cox model were age, BMI, waist circumference, gender, weight categories, arterial hypertension, diabetes mellitus (DM), previous CVA, atherogenic index, smoking status, previous MI, chronic obstructive pulmonary disease (COPD), type of revascularization (PCI/CABG), persistent/permanent atrial fibrillation (AF), initial LVEF, peripheral vascular disease, Charlson comorbidity index (CCI) (https://www.mdcalc.com/calc/3917/charlson-comorbidity-index-cciprimary) [27, 28], initial SS. The diagnostic significance of BMI was assessed using receiver-operating characteristic (ROC) curves. All calculations were executed using SPSS Statistics software 23.0 (IBM Corporation, Armonk, New York, USA), and the value of p < 0.05 was considered statistically significant.

## Results

### Baseline characteristics

Our study analyzed 406 patients, categorized based on BMI levels into (1) normal weight (11.8%, n = 48), (2) overweight (41.6%, n = 169), and (3) obese (46.6%, n = 189). There were no patients with a BMI less than 18.5 kg/m2 in our study. The initial characteristics are presented in Table 1. Women (n = 70/17.2%) were more likely to be obese patients (64.3%) than normal (10%) and overweight ones (25.7%) (*p* = 0.04). On the other hand, overweight (44.9%) and obese (42.9%) male patients outnumbered those who were normal weight (12.2%), at *p* = 0.04, among men (n = 336/82.8%). The atherogenic index (AI) applied in this study is derived by applying the formula: (total cholesterol minus high-density lipoproteins) divided by high-density lipoproteins. AI was higher in the overweight (3.8 [2.8–4.7]) and obese (3.7 [2.7–5]) groups, compared with patients with normal weight (3.2 [1.8–4]), at *p* = 0.013. DM and high-grade arterial hypertension (AH) were more prevalent in obese patients than in normal or overweight ones (p < 0.0001 for both indicators). Concerning other initial characteristics, patients with different weight categories did not differ.

**Table 1:** Baseline characteristics

	Normal (n = 48/11.8%)	Overweight (n = 169/41.6%)	Obesity (n = 189/46.6%)	*p *value
Age, years	55.3 ± 6.2	55.5 ± 6.6	55.7 ± 5.9	0.95
Women	7 (14.6%)	18 (10.7%)	45 (23.8%)	0.04
Men	41 (85.4%)	151 (89.3%)	144 (76.2%)	0.04
Family history of heart disease	12 (25%)	37 (21.9%)	58 (30.7%)	0.17
History of smoking	18 (37.5%)	55 (32.5%)	60 (31,7%)	0.75
Waist circumference, male	87 (83–90.5)	100.5 (96.75–104)	108.5 (103–115)	< 0.0001
Waist circumference, female	88 (81.5–91.5)	102 (90–110)	106.5 (101.5–116.5)	0.006
Dyslipidemia	32 (66.7%)	141 (83.4%)	151 (79.9%)	0.05
Atherogenic index	3.2 (1.8–4)	3.8 (2.8–4.7)	3.7 (2.7–5)	0.013
GFR, ml/min/1.73m2	88.3 (± 17)	89.9 (± 17.3)	91.9 (± 21)	0.43
Diabetes mellitus	8 (16.7%)	46 (27.2%)	80 (42.3%)	< 0.0001
Hypertension	45 (93.8%)	165 (97.6%)	189 (100%)	0.002
Degrees of hypertension				< 0.0001
Mild hypertension	6 (12.5%)	11 (6.5%)	3 (1.6%)	
Moderate hypertension	15 (31.3%)	61 (36.1%)	53 (28%)	
Severe hypertension	24 (50%)	93 (55%)	133 (70.4%)	
Previous myocardial infarction	28 (58.3%)	112 (66.3%)	114 (60.3%)	0.4
Previous CVA (stroke or transient ischaemic attack)	4 (8.3%)	13 (7.7%)	13 (6.9%)	0.7
Atrial fibrillation	7 (14.6%)	28 (16.6%)	45 (23.8%)	0.14
Peripheral arterial disease	10 (20.8%)	24 (14.2%)	34 (18%)	0.46
Chronic obstructive pulmonary disease	5 (10.4%)	18 (10.7%)	27 (14.3%)	0.53
Charlson Comorbidity Index [27, 28]	4.5 (± 2)	4.76 (± 2.2)	4.82 (± 1.8)	0.6
Left ventricular ejection fraction (%)	54.6 (± 6.4)	55.7 (± 6.6)	54 (± 6.6)	0.12
SYNTAX Score, mean	20.4 (± 7.4)	21.2 (± 6.6)	20.5 (± 6.8)	0.6
Disease extent				0.5
Two-vessel disease	28 (58.3%)	83 (49.1%)	95 (50.3%)	
Three-vessel disease	20 (41.7%)	86 (50.9%)	94 (49.7%)	
Type of revascularization				0.39
PCI	22 (45.8%)	78 (46.2%)	100 (52.9%)	
CABG	26 (54.2%)	91 (53.8%)	89 (47.1%)	

Values are shown as mean ± SD (n), Me(Q1-Q3) or % (n/N)

CABG = coronary artery bypass grafting; CVA = cerebrovascular accident; PCI = percutaneous coronary intervention; Atherogenic index (AI) was calculated using the formula AI = (total cholesterol - high-density lipoproteins)/high-density lipoproteins; GFR = glomerular filtration rate according to the Cockcroft-Gault formula

### Outcomes

Clinical outcomes based on weight gradations are presented in Tables 2 and 3. By employing the weight gradation, overweight patients had a lower risk of cardiac death compared to obese patients (8.3% vs. 15.9%, HR 0.5, CI 0.2–0.96, *p* = 0.037, respectively). On average, SS were in dynamics, which were higher in patients with obesity and overweight, compared with patients with normal weight (25 [17–33.5], 23 [11.3–32.5], and 16.3 [7.3–26.4], *p* = 0.026, respectively). When analyzing outcomes among obesity groups (I, II, and III Classes), obese Class I patients had a lower risk of requiring repeated revascularization (HR 0.5, 95% CI 0.25–0.97, *p* = 0.04), cardiac death (HR 0.32, 95% CI 0.12–0.88, *p* = 0.028) and MI (HR 0.29, 95% CI 0.11–0.74, *p* = 0.01), in relation to obese Class III patients. For other events, the groups with different classes of obesity did not have significant differences.

**Table 2:** Long-term outcomes by level of BMI

	Normal (n = 48/11.8%)	Overweight (n = 169/41.6%)	Obesity (n = 189/46.6%)	Hazard ratio (95% CI) normal/obesity, overweigt/obesity	*p *value	*p *value interaction
MACCE	28 (58.3%)	96 (56.8%)	133 (70.4%)	0.76 (0.5–1.14)	0.19	0.22
				0.82 (0.63–1.1)	0.15	
Repeat revascularization	18 (37.5%)	67 (39.6%)	97 (51.3%)	0.65 (0.39–1.1)	0.09	0.12
				0.78 (0.57–1.06)	0.12	
All-cause-death /MI/Stroke/TIA	19 (39.6%)	59 (34,9%)	71 (37.6%)	1.06 (0.64–1.75)	0.84	0.86
				0.93 (0.66–1.3)	0.68	
Death, all-cause	11 (22.9%)	25 (14.8%)	41 (21.7%)	1.03 (0.53–2.0)	0.92	0.24
				0.67 (0.41–1.1)	0.11	
Cardiac death	6 (12.5%)	14 (8.3%)	30 (15.9%)	0.77 (0.32–1.86)	0.56	0.11
				0.51 (0.27–0.96)	0.037	
Non-cardiac death	5 (10.4%)	11 (6.5%)	11 (5.8%)	1.74 (0.61–5.0)	0.3	0.58
				1.1 (0.48–2.53)	0.83	
Myocardial infarction	5 (10.4%)	22 (13%)	29 (15.3%)	0.69 (0.27–1.78)	0.44	0.65
				0.82 (0.47–1.43)	0.48	
Stroke/TIA	5 (10.4%)	23 (13.6%)	24 (12.7%)	0.83 (0 .32–2.17)	0.7	0.85
				1.1 (0.6–1.9)	0.78	
LVEF during follow-up (%)*	52.9 (± 9.8)	51.8 (± 10,3)	50 (± 10.9)		0.28	
Diminution in LVEF	8 (23.5%)	40 (32.3%)	66 (39.8%)	0.5 (0.24–1.05)	0.07	0.15
				0.82 (0.55–1.2)	0.32	
Heart chambers dilatation + valvular insufficiency	4 (11.8%)	18 (14.5%)	30 (18.1%)	0.58 (0.2–1.65)	0.3	0.54
				0.83 (0.46–1.48)	0.52	
SYNTAX Score during follow-up*	16.3 (7.3–26.4)	23 (11.3–32.5)	25 (17–33.5)		0.024	
SYNTAX Score, ≥ 33 during follow-up	3 (12.5%)	20 (26%)	39 (28.5%)	0.37 (0.11–1.19)	0.096	0.24
				0.86 (0 .5–1.47)	0.58	
Left main disease during follow-up	2 (8.3%)	3 (3.8%)	9 (6.6%)	1.14 (0.25–5.3)	0.87	0.6
				0.54 (0.15–1.99)	0.35	

Values are number of events (%), unless otherwise indicated

^*^Values are shown as mean ± SD (n), Me(Q1-Q3) or % (n/N)

BMI = body mass index; CABG = coronary artery bypass grafting; PCI = percutaneous coronary intervention; MACCE-major adverse cardiac and cerebrovascular events = All-cause-death + MI + Stroke/TIA + Repeat revascularisation; MI = myocardial infarction; TIA = transient ischemic attack; LVEF = Left ventricular ejection fraction; HR = Hazard Ratio; CI = Confidence interval

**Table 3:** Long-term outcomes according to obesity categories

	Obese, Cl. I (n = 122/ 64.6%)	Obese, Cl. II (n = 51/ 27%)	Obese, Cl. III (n = 16/8.5%)	Hazard ratio (95% CI)	*p *value	*p* value interaction
				Obese, Cl. I/III,		
				Obese, Cl. II/III		
MACCE	86 (70.5%)	34 (66.7%)	13 (81.3%)	0.61 (0.34–1.1)	0.09	0.25
				0.65 (0.34–1.23)	0.18	
Repeat revascularization	66 (54.1%)	21 (41.2%)	10 (62.5%)	0.5 (0.25–0.97)	0.04	0.11
				0.48 (0.22–1.02)	0.055	
All-cause-death /MI/Stroke/TIA	42 (34.4%)	21 (41.2%)	8 (50%)	0.58 (0.27–1.24)	0.16	0.31
				0.74 (0.33–1.67)	0.46	
Death, all-cause	20 (16.4%)	16 (31.4%)	5 (31.3%)	0.42 (0.16–1.11)	0.08	0.045
				0.88 (0.32–2.39)	0.79	
Cardiac death	15 (12.3%)	10 (19.6%)	5 (31.3%)	0.32 (0.12–0.88)	0.028	0.07
				0.56 (0.19–1.64)	0.29	
Non-cardiac death	5 (4.1%)	6 (11.8%)	0	7259 (0.000–1.55)	0.94	0.16
				23,127 (0.000—4.9)	0.94	
Myocardial infarction	16 (13.1%)	7 (13.7%)	6 (37.5%)	0.29 (0.11–0.74)	0.01	0.03
				0.34 (0.11–1.0)	0.05	
Stroke/TIA	17 (13.9%)	5 (9.8%)	2 (12.1%)	0.99 (0.23–4.33)	0.99	0.82
				0.73 (0.14–3.75)	0.71	
LVEF during follow-up (%)*	50.97 (± 10.5%)	48.6 (± 11.7)	50.6 (± 12.1)		0.48	
Diminution in LVEF	40 (36.7%)	20 (46.5%)	6 (42.9%)	0.49 (0.21–1.19)	0.12	0.09
				0.82 (0.33–2.05)	0.67	
Heart chambers dilatation + valvular insufficiency	15 (13.8%)	13 (30.2%)	2 (14.3%)	0.58 (0.13–2.57)	0.48	0.04
				1.53 (0.34–6.79)	0.58	
SYNTAX score during follow-up*	24.6 (± 14.5)	25.4 (± 12.5)	29 (± 13)		0.59	
SYNTAX Score, ≥ 33 during follow-up	26 (28.6%)	9 (26.5%)	4 (33.3%)	0.55 (0.19–1.59)	0.27	0.54
				0.62 (0.19–2.04)	0.44	
Left main disease during follow-up	6 (6.6%)	2 (5.9%)	1 (8.3%)	0.55 (0.06–4.64)	0.58	0.85
				0.68 (0.06–7.69)	0.76	

Values are number of events (%), unless otherwise indicated

^*^Values are shown as mean ± SD (n), Me(Q1-Q3) or % (n/N)

CABG = coronary artery bypass grafting; PCI = percutaneous coronary intervention; MACCE-major adverse cardiac and cerebrovascular events = All-cause-death + MI + Stroke/TIA/PE + Repeat revascularisation; MI = myocardial infarction; TIA = transient ischemic attack; LVEF = Left ventricular ejection fraction; HR = Hazard Ratio; CI = Confidence interval; Cl. = Class

**Fig. 1:**
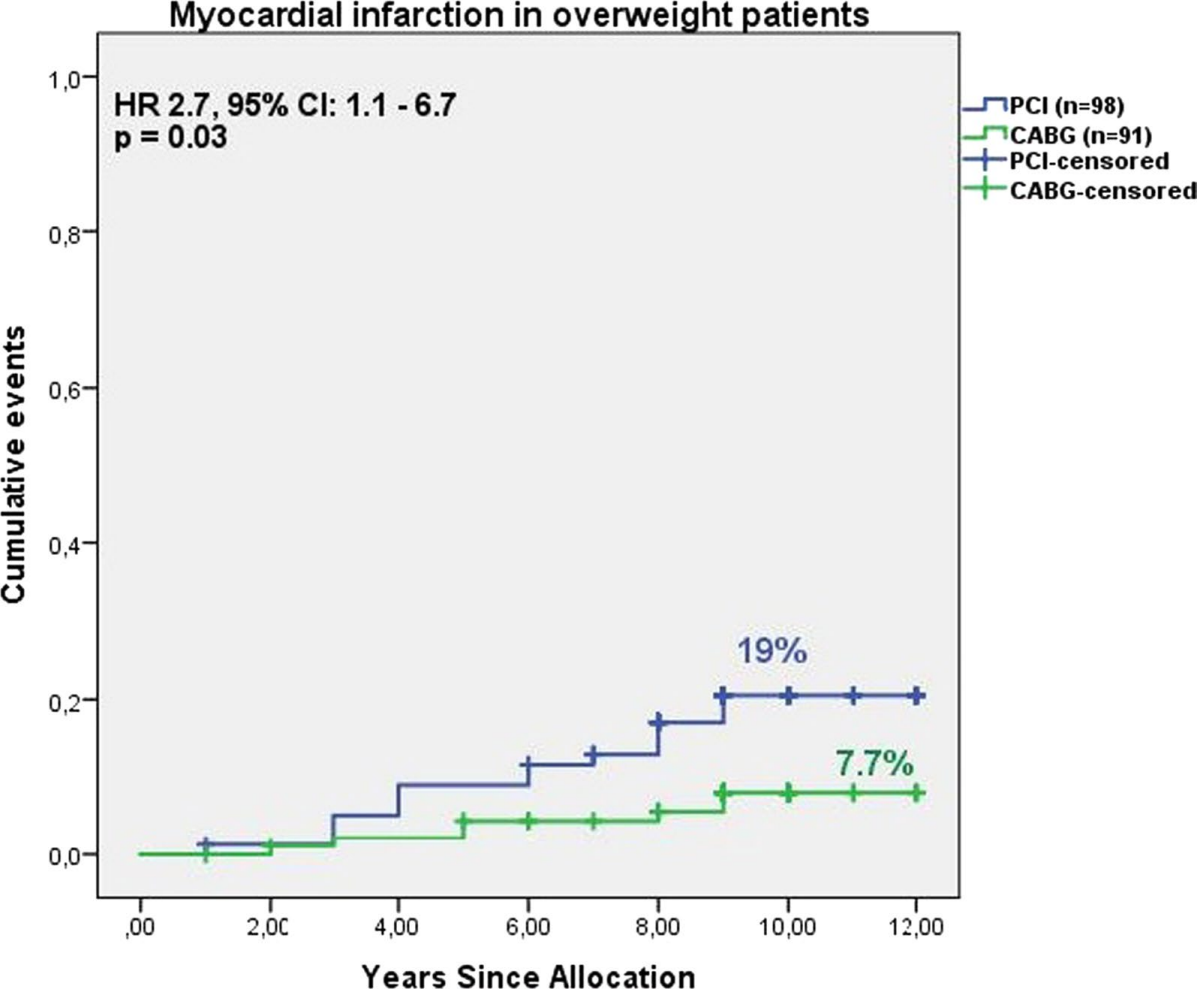
Nine-year Kaplan–Meier curves for Myocardial infarction according to revascularization treatment in overweight patients. CABG = coronary artery bypass graft surgery; PCI = percutaneous coronary intervention; HR = Hazard Ratio; CI = Confidence interval

There were no differences between the revascularization outcomes for the normal weight group based on the strategy (Table 4). For overweight patients, the probability of MI development after PCI was higher than after CABG (19% vs. 7.7%, HR 2.7, 95% CI 1.1–6.7, *p* = 0.03) (Fig. 1). Both overweight and obese patients showed a higher risk of repeated revascularization following PCI as opposed to CABG (59% vs. 23%, HR 2.7, 95% CI 1.6–4.55, p < 0.001; and 67% vs. 33.7%, HR 1.99, 95% CI 1.3–3.1, *p* = 0.002, respectively); the likelihood of a high degree of coronary artery lesion (SS ≥ 33) after PCI was less than after CABG (9.8% vs. 44.4%, HR 0.23, 95% CI 0.08—0.7, *p* = 0.009 and 12.8% vs. 49%, HR 0.28, 95% CI 0.14–0.57, *p* = 0.001, respectively) (Table 4).

**Table 4:** Long-term outcomes according to BMI and revascularization treatment

	Normal (n = 48/11.8%)		Hazard ratio (95% CI)	*p *value	Overweight (n = 169/41.6%)		Hazard ratio (95% CI)	*p *value	Obesity (n = 189/46.6%)		Hazard ratio (95% CI)	*p *value	*p* value interaction
	PCI (22/45.8%)	CABG (26/54.2%)			PCI (78/46.2%)	CABG (91/53.8%)			PCI (100/52.9%)	CABG (89/47%)			
MACCE	16 (72.7%) -	12 (46.2%)	1.74 (0.81–3.74)	0.16	55 (70.5%)	41 (45.1%)	1.48 (0.98–2.23)	0.06	78 (78%)	55 (61.8%)	1.36 (0.96–1.9)	0.08	0.004
Repeat revascularization	11 (50%)	7 (26.9%)	1.97 (0.74—5.24)	0.18	46 (59%)	21 (23.1%)	2.69 (1.6–4.55)	< 0.0001	67 (67%)	30 (33.7%)	1.99 (1.29–3.07)	0.002	< 0.0001
All-cause-death /MI/Stroke/TIA	10 (45.5%)	9 (34.6%)	1.5 (0.62–3.79)	0.36	27 (34.6%)	32 (35.2%)	0.99 (0.59–1.67)	0.98	36 (36%)	35 (39.3%)	0.95 (0.59–1.52)	0.84	0.91
Death, all-cause	6 (27.3%)	5 (19.2%)	1.8 (0.55–5.99)	0.33	11 (14.1%)	14 (15.4%)	0.98 (0.44–2.16)	0.96	18 (18%)	23 (25.8%)	0.7 (0.38–1.3)	0.26	0.63
Cardiac death	4 (18.2%)	2 (7.7%)	3 (0.54–16.6)	0.21	4 (5.1%)	10 (11%)	0.48 (0.15–1.53)	0.21	12 (12%)	18 (20.2%)	0.59 (0.29–1.24)	0.165	0.22
Myocardial infarction	1 (4.5%)	4 (15.4%)	0.29 (0.03–2.56)	0.26	15 (19.2%)	7 (7.7%)	2.73 (1.1–6.7)	0.029	19 (19%)	10 (11.2%)	1.7 (0.79–3.67)	0.17	0.04
Stroke/TIA	4 (18.2%)	1 (3.8%)	5.98 (0.66–54.2)	0.11	8 (10.3%)	15 (16.5%)	0.59 (0.25–1.4)	0.22	12 (12%)	12 (13.5%)	0.9 (0.41—2)	0.8	0.64
Diminution in LVEF	3 (21.4%)	5 (25%)	0.79 (0.18–3.5)	0.76	16 (27.1%)	24 (36.9%)	0.77 (0.4–1.46)	0.43	27 (30.3%)	39 (50.6%)	0.62 (0.38–1.01)	0.055	0.055
Heart chambers dilatation + valvular insufficiency	2 (14.3%)	2 (10%)	1.84 (0.26–13.25)	0.55	7 (11.9%)	11 (16.9%)	0.75 (0.29–1.9)	0.54	11 (12.4%)	19 (24.7%)	0.55 (0.26–1.15)	0.11	0.18
SYNTAX Score, ≥ 33, during follow-up	0	3 (21.4%)	0.01 (0.000–229)	0.39	4 (9.8%)	16 (44.4%)	0.23 (0.08–0.69)	0.009	10 (12.8%)	29 (49.2%)	0.28 (0.14–0.57)	0.001	< 0.0001
Left main disease during follow-up	0	2 (14.3%)	0.02 (0.000–1708)	0.49	1 (2.4%)	2 (5.4%)	0.49 (0.05–5.48)	0.57	5 (6.4%)	4 (6.8%)	0.92 (0.25–3.45)	0.9	0.38

Values are number of events (%), unless otherwise indicated

BMI = body mass index; CABG = coronary artery bypass grafting; PCI = percutaneous coronary intervention; MACCE-major adverse cardiac and cerebrovascular events = All-cause-death + MI + Stroke/TIA + Repeat revascularisation; MI = myocardial infarction; TIA = transient ischemic attack; LVEF = Left ventricular ejection fraction; HR = Hazard Ratio; CI = Confidence interval

Further analysis within obesity classes showed that first-class obesity patients had a higher risk of needing repeated revascularization following PCI as opposed to CABG (74.6% vs. 32.2%, HR 1.93, 95% CI 1.1–3.3, *p* = 0.02). At the same time, the probability of progression of atherosclerotic lesions of the coronary arteries (SS ≥ 33) after PCI was less than after CABG (11.5% vs. 51.3%, HR 0.2, 95% CI 0.08–0.5, *p* = 0.001). For patients with second and third classes of obesity, we found no differences in revascularization outcomes depending on the strategy (Table 5).

**Table 5:** Long-term outcomes according to obesity categories and revascularization treatment

	Obese, Cl. I (n = 122/64.6%)		Hazard ratio (95% CI)	*p *value	Obese, Cl. II (n = 51/27%)		Hazard ratio (95% CI)	*p *value	Obese, Cl. III (n = 16/8,5%)		Hazard ratio (95% CI)	*p *value	*p* value interaction
	PCI (63/51.6%)	CABG (59/48,4%)			PCI (24/47.1%)	CABG (27/52.9%)			PCI (13/81.3%)	CABG (3/18.8%)			
MACCE	51 (81%)	35 (59,3%)	1.32 (0.85–2.03)	0.21	16 (66.7%)	18 (66.7%)	1.26 (0.63–2.5)	0.51	11 (84.6%)	2 (66.7%)	1.67 (0.36–7.8)	0.5	0.08
Repeat revascularization	47 (74.6%)	19 (32.2%)	1.93 (1.13 -3.29)	0.017	11 (45.8%)	10 (37%)	1.84 (0.76–4.47)	0.18	9 (69.2%)	1 (33.3%)	2.95 (0.36–24.2)	0.3	0.002
All-cause-death /MI/Stroke/TIA	22 (34.9%)	20 (33.9%)	1.02 (0.56–1.87)	0.95	8 (33.9%)	13 (48.1%)	0.82 (0.33–2)	0.66	6 (46.2%)	2 (66.7%)	0.76 (0.15–3.76)	0.7	0.84
Death, all-cause	7 (11.1%)	13 (22%)	0.47 (0.19–1.17)	0.1	7 (29.2%)	9 (33.3%)	1.15 (0.42–3.18)	0.79	4 (30.8%)	1 (33.3%)	1.05 (0.12–9.4)	0.96	0.26
Cardiac death	4 (6.3%)	11 (18.6%)	0.32 (0.1–1.02)	0.053	4 (16.7%)	6 (22.2%)	1.1 (0.28–3.97)	0.93	4 (30.8%)	1 (33.3%)	1.05 (0.12–9.4)	0.96	0.165
Myocardial infarction	11 (17.5%)	5 (8.5)	1.96 (0.68–5.66)	0.21	3 (12.5%)	4 (14.8%)	1.23 (0.25–6.1)	0.8	5 (38.5%)	1 (33.3%)	1.21 (0.14–10.4)	0.86	0.17
Stroke/TIA	9 (14.3%)	8 (13.6%)	0.98 (0.38–2.54)	0.96	2 (8.3%)	3 (11.1%)	0.84 (0.14–5.1)	0.85	1 (7.7%)	1 (33.3%)	0.27 (0.02–4.4)	0.36	0.8
Diminution in LVEF	18 (31%)	22 (43.1%)	0.64 (0.34–1.2)	0.17	5 (25%)	15 (65.2%)	0.53 (0.19–1.48)	0.23	4 (36.4%)	2 (66.7%)	0.6 (0.11–3.3)	0.56	0.055
Heart chambers dilatation + valvular insufficiency	7 (12.1%)	8 (15.7%)	0.77 (0.28–2.13)	0.62	2 (10%)	11 (47.8%)	0.27 (0.06–1.21)	1.09	2 (18.2%)	0	28 (0.000–114,856,747)	0.67	0.11
SYNTAX Score, ≥ 33 during follow-up	6 (11.5%)	20 (51.3%)	0.2 (0.08–0.51)	0.001	2 (12.5%)	7 (38.9%)	0.36 (0.08–1.7)	0.2	2 (20%)	2 (100%)	0.4 (0.05–3.1)	0.38	0.001
Left main disease during follow-up	3 (5.8%)	3 (7.7%)	0,55 (0.11—2.77)	0.47	1 (6.3%)	1 (5.6%)	1.13 (0.07–8.12)	0.93	1 (10%)	0	31.5 (0.000–7,314,677,856)	0.73	0.9

Values are number of events (%), unless otherwise indicated

CABG = coronary artery bypass grafting; PCI = percutaneous coronary intervention; MACCE-major adverse cardiac and cerebrovascular events = All-cause-death + MI + Stroke/TIA + Repeat revascularisation; MI = myocardial infarction; TIA = transient ischemic attack; LVEF = Left ventricular ejection fraction; HR = Hazard Ratio; CI = Confidence interval; Cl. = Class

The development of adverse events was performed using univariate and multivariate Cox regression to find independent predictors. The following indicators were utilized as predictors: gender, age, smoking, type of revascularization (CABG/PCI), BMI, previous CVA, peripheral atherosclerotic vascular disease, hypertension, AF, weight gradations (normal, overweight, and obesity), DM, classes of obesity, initial LVEF, waist circumference, COPD, atherogenic index, previous MI, and initial SS. Consequently, for all revascularized patients BMI was linked to the risk of all-cause mortality (HR 1.05, 95% CI 1.002–1.11, *p* = 0.04), and cardiac death (HR 1.1, 95% CI 1.02–1.15, *p* = 0.01). Class III obesity (BMI ≥ 40), compared to normal weight (BMI = 18.5–24.9), was associated with the risk of CHF with reduced LVEF (HR 3.2, 95% CI 1.1–9.3, *p* = 0.03), and repeated revascularization (HR 2.9, 95% CI 1.4–6.5, *p* = 0.06). It should be noted that in a multivariate analysis conducted separately for the PCI and CABG groups, Obesity Class III among stented patients was associated with an increased risk of repeat revascularization (HR 2.5, 95% CI 1.02–6, *p* = 0.044). Additionally, for surgically treated patients, a BMI exceeding 29 kg/m2 was associated with a risk of developing CHF with reduced LVEF (HR 1.06, 95% CI 1.01–1.12, *p* = 0.015). Yet, BMI did not significantly impact other study endpoints (Table 6).

**Table 6:** Results of multivariate analysis for BMI

Events	Unadjusted HR (95% CI)	*p* value	Adjusted HR (95% CI) *	*p* value
All-cause-death /MI/CVA	1.01 (0.98–1.046)	0.52	–	–
Death, all-cause	1.06 (1.01–1.11)	0.01	1.05 (1.002–1.11)	0.04
Cardiac death	1.1 (1.04–1.16)	0.002	1.1 (1.02–1.15)	0.01
Myocardial infarction	1.05 (0.99–1.11)	0.078	–	
Stroke/TIA	0.98 (0.9–1.05)	0.66	–	
Heart failure with decrease in LVEF	1.06 (1.02–1.1)	0.004	1.01 (0.9–1.09)	0.77
Heart failure with heart chambers dilatation and valvular insufficiency	1.07 (1.01–1.14)	0.015	1.06 (0.99–1.12)	0.07
SYNTAX Score, ≥ 33, during follow-up	1.03 (0.97–1.09)	0.3	–	
Repeat revascularization	1.04 (1.01–1.07)	0.02	1.04 (0.97–1.1)	0.25

^*^Adjusted for gender, age, smoking status, dyslipidemia, arterial hypertension, diabetes mellitus, previous myocardial infarction, previous CVA, peripheral vascular disease, atrial fibrillation, COPD, CCI, primary LVEF, type of revascularization (PCI/CABG), initial SYNTAX score

CABG = coronary artery bypass grafting; CCI = Charlson Comorbidity Index; CI = confidence interval COPD = Chronic obstructive pulmonary disease; CVA = cerebrovascular accident; HR = Hazard ratio; MI = myocardial infarction; PCI = percutaneous coronary intervention; TIA = transient ischemic attack; LVEF = Left ventricular ejection fraction

**Fig. 2:**
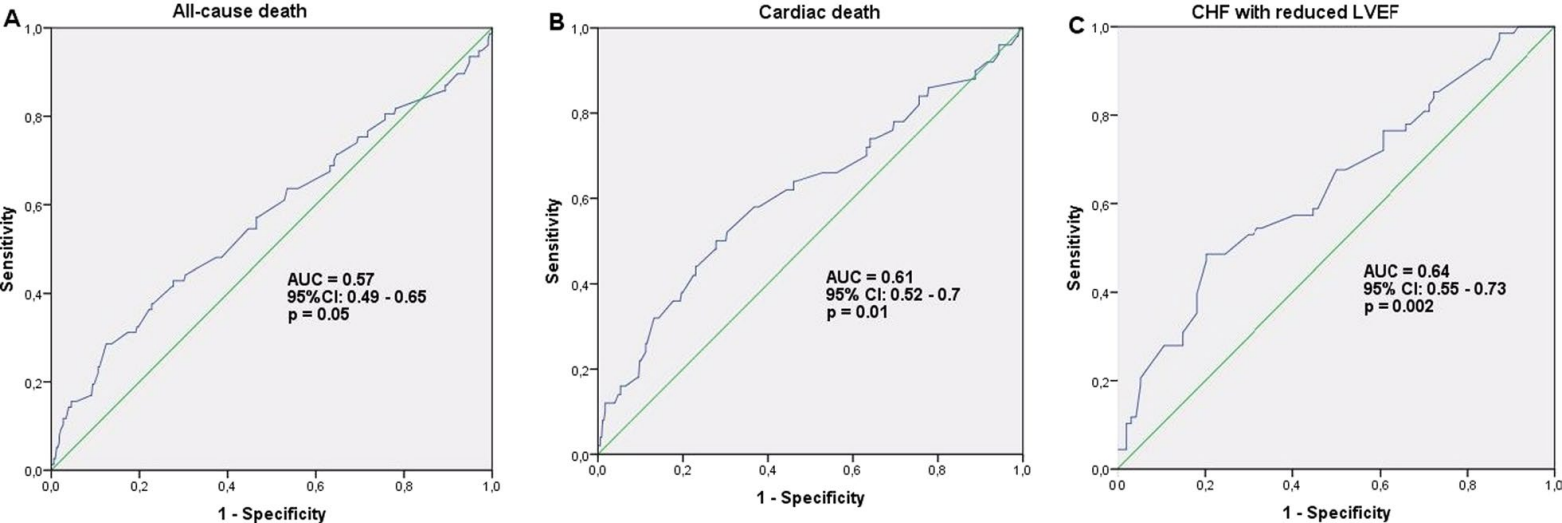
ROC curves for BMI. Receiver-operating characteristic (ROC) curves for (**A**) All-cause-Death; (**B**) Cardiac death; and (**C**) CHF with reduced LVEF based on the BMI are shown. (**A**), (**B**): Overall population; (**C**): CABG cohort. AUC = Area under curve; BMI = body mass index; CABG = coronary artery bypass grafting; CHF = chronic heart failure; CI = confidence interval; LVEF = left ventricular ejection fraction

A Receiver Operating Characteristic (ROC) analysis was performed for evaluating the diagnostic significance, sensitivity, and specificity of BMI. The area under the ROC curve (AUC) demonstrated that BMI had fail predictive capability for all-cause mortality (AUC 0.57, 95% CI 0.49–0.65, *p* = 0.05). The AUC of the ability of BMI to predict cardiac death reached 0.61 (95% CI 0.52–0.7, *p* = 0.01), demonstrating poor model quality. Also for patients, who underwent surgery, the AUC of the ability of BMI to predict the development of CHF with reduced LVEF was 0.64 (95% CI 0.55–0.73, *p* = 0.002), indicating a poor model quality too (Fig. 2).

## Discussion

The body's excessive or abnormal fat or adipose tissue accumulation, referred to as obesity, deteriorates health [26]. The tool used to assess obesity is a BMI ≥ 30 kg/m^2^ [25, 26]. BMI assessment has limitations due to its inability to describe the relative contribution of adipose tissue, muscle mass, and bone mass [29, 30]. Other indicators of central obesity involve waist-to-hip ratio, waist-to-height ratio, and waist circumference [26, 31]. Due to the retrospective nature of our study, we initially had data only on BMI and waist circumference. Accordingly, we were able to assess changes only in these measurements over time. Other assessments of obesity were not applied in our observation. The measure of waist circumference in our analysis did not show a significant impact on revascularization outcomes.

Obesity is the most important factor in the development of CAD [3, 4]. In the context of the global obesity epidemic, multivessel disease (MVD) is reasonably represented among patients with CAD [7, 32]. Thus, in general, the prevalence of MVD ranges from 30 to 40% of patients with CAD, and among patients with acute coronary syndrome MVD occurs in about 50% of patients [32, 33]. Despite advances in PCI, CABG offers more comprehensive revascularization for more compound multivessel coronary artery disease [34]. It should be noted that surgery is still more invasive treatment method than PCI and it is associated with more pronounced technical difficulties, wound complications and postoperative respiratory problems in this cohort of patients [35]. To objectively compare the outcomes of PCI and CABG, we excluded patients with severe coronary lesions who had clear indications for surgery. Only patients with low and intermediate coronary lesions (SYNTAX score < 33), who were eligible for both PCI and CABG, were included in the analysis. Taking into account the growth of the overweight and obese population, the selection of the optimal revascularization method for this group of patients, and determining the influence of weight categories on the results of PCI and CABG, are of clinical interest and debate.

Despite the existence of a proven causal relationship between morbid obesity and increased cardiovascular morbidity [3, 4], researchers have observed the "obesity paradox," noting that obesity can have a protective effect on postoperative complications and mortality in patients undergoing surgery or interventional treatments [14, 16–18, 21]. Even so, some researchers have critically evaluated the "obesity paradox" due to the presence of a selection bias in observations, exclusion of early death cases from long-term studies, lack of proper consideration of unintentional weight loss due to a high level of comorbidity, younger patients with obesity, short-term observation period, insufficient consideration of distorting factors, and association of smoking with lower body weight [30, 36, 37]. In our study, obesity did not express "protective" properties by the main MACCE. Previous long-term studies comparing PCI and CABG outcomes have shown the superiority of surgery in MACCE [8–10]. In our study, for overweight patients, CABG also demonstrated superiority over PCI in terms of the risk of developing MI. According to other adverse events, the strategies did not show advantages across all weight categories.

In many other observations, PCI is associated with the likelihood of repeated revascularization [8, 9, 11]. In our study, PCI was also associated with the risk of repeated revascularization among overweight and Class I obese patients (HR 2.7, 95% CI 1.6–4.55, p < 0.0001 and HR 1.9, 95% CI 1.1–3.3, *p* = 0.017, respectively). In other weight categories, the association between PCI and repeated revascularization was also observed but without statistical significance (Tables 4, 5). It is noteworthy that Obesity Class 3 in stented patients was associated with an increased risk of repeat revascularization, consistent with findings from previous studies [38].

In our observation, for individuals with overweight and Class I obesity, CABG was associated with the risk of coronary atherosclerosis progression (SS > 32) [HR 4.4, 95% CI 1.5–13, *p* = 0.009 and HR 4.9, 95% CI 1.9–12, *p* = 0.001, correspondingly]. In other weight categories, high-grade coronary artery lesions (SS > 32) also developed more frequently after CABG than after PCI, but the data were not statistically significant (Tables 4,5). This can likely be attributed to the propensity of grafts, particularly vein grafts, to undergo remodeling, atherosclerosis, and progressive intimal hyperplasia, leading to occlusion or graft stenosis [39, 40]. Graft patency is a critical factor influencing long-term survival and clinical prognosis following CABG [40, 41]. The use of the saphenous vein graft (SVG) is widespread and accounts for more than 80% of CABG cases worldwide [40, 42]. SVG in CABG are characterized by a high incidence of early atherosclerosis, intimal hyperplasia, and thrombosis, resulting in graft failure in 12–20% of cases within a year [40, 43] and 50–60% within a decade [40, 44]. Meanwhile, advances in interventional treatment achieve success in reducing in-stent restenosis. Previous studies indicate that bare-metal stents (BMS) lowered the incidence of restenosis comparing balloon angioplasty from 30–60% to 15–40%, and DES further reduced this rate by up to 15% [45].

Thus, through careful preoperative planning, advanced surgical and interventional techniques, and improved postoperative care, healthcare providers can optimize treatment outcomes for obese patients undergoing coronary revascularization. A detailed understanding of the impact of obesity on PCI and CABG and a personalized approach to patient management can enhance the success of procedures and long-term survival. Addressing specific issues related to different weight categories remains a crucial component of optimizing care for patients undergoing coronary revascularization.

### Study limitations

Our results of the study should be considered with the following limitations in mind.

Firstly, the modest sample size may limit the statistical power of this analysis.

Secondly, despite implementing various measures and corrections, the retrospective observational nature of the study introduces the potential for systematic selection bias.

Thirdly, the study's cohort consisted of stable multivessel CAD patients, without left main disease, who had low and intermediate SS and received primary PCI or CABG prior to age 65. As a result, these outcomes are not applicable to other CAD populations.

Fourthly, it is important to note that patients underwent PCI with DES and surgical procedures corresponding to the guidelines from 2010–2013. Thus, our findings may not be fully applicable to modern treatment technologies. Long-term observations, while valuable, are inevitably based on somewhat outdated technologies.

## Conclusions

Thus, there were no significant weight-related impacts on long-term outcomes among patients who underwent surgery. Whereas in stented patients, Class III obesity was associated with the risk of repeat revascularization. When comparing PCI and CABG, for overweight and Class I obesity patients, CABG was associated with a likelihood of coronary atherosclerosis progression (SS ≥ 33), while PCI was linked to the risk of repeat revascularization. For overweight patients, CABG outperformed PCI in terms of the risk of MI. For other adverse events in patients of different weight categories, PCI and CABG did not reveal any significant benefits.

## Data Availability

Data confirming the results of this study are stored in the databases of the National Research Cardiac Surgery Center (Astana), Pavlodar Regional Cardiology Center, and the national polyclinic and inpatient registers of the Kazakhstan Republican e-Health Center. This data is not publicly available. Nonetheless, acceptable information can be provided by the authors upon reasonable request and with the permission of the directors of these centers.

## Abbreviations

AI: Atherogenic index
AH: Arterial hypertension
AF: Atrial fibrillation
AUC: Area under curve
BMI: Body mass index
CAD: Coronary artery disease
CABG: Coronary artery bypass grafting
CHF: Chronic heart failure
CI: Confidence interval
CCI: Charlson comorbidity index
CVD: Cardiovascular diseases
CVA: Cerebrovascular accident
DES: Drug-eluting stent
Dg: Degree
GFR: Glomerular filtration rate
HR: Hazard ratio
LVEF: Left ventricular ejection fraction
MACCE: Major adverse cardiac and cerebrovascular events
MI: Myocardial infarction
MVD: Multivessel disease
PCI: Percutaneous coronary intervention
RCT: Randomized clinical trials
ROC: Receiver operating characteristics
SS: SYNTAX score
SVG: Saphenous vein graft
TIA: Transient ischemic attack
